# The Effect of *Carvacrol* on Lipid Profiles, Oxidative Stress, Inflammatory Markers, and Renal Injury in STZ‐Induced Diabetic Rats

**DOI:** 10.1002/fsn3.71615

**Published:** 2026-06-02

**Authors:** Vida Seyedmousavi Dijvejin, Vahid Asghariazar, Helda Tutunchi, Elnaz Faramarzi, Hamed Shoorei, Rouhallah Moradpour, Mehdi Asghari Vostakolaei

**Affiliations:** ^1^ Cancer Immunology and Immunotherapy Research Center Ardabil University of Medical Sciences Ardabil Iran; ^2^ Endocrine Research Center Tabriz University of Medical Sciences Tabriz Iran; ^3^ Department of Chemical and Biochemical Engineering University of Western Ontario London Ontario Canada; ^4^ Medical Radiation Sciences Research Center Tabriz University of Medical Sciences Tabriz Iran; ^5^ Department of Anatomical Sciences, Faculty of Medicine Tabriz University of Medical Sciences Tabriz Iran; ^6^ Clinical Research Development Unit of Tabriz Valiasr Hospital Tabriz University of Medical Sciences Tabriz Iran; ^7^ Cellular and Molecular Research Center Birjand University of Medical Sciences Birjand Iran

**Keywords:** carvacrol, diabetes, glycemic indices, inflammation, lipid profile, oxidative stress

## Abstract

The present experimental study aimed to examine the effects of carvacrol on glycemic control, lipid profile, oxidative stress, inflammatory markers, and renal injury markers (glomerular diameters, urinary space diameters, and number of glomeruli) in diabetic rats. Animals (*n* = 30) were randomly divided into three groups: control group, diabetic group (induced by STZ: 50 mg/kg), and 250 mg/kg carvacrol–treated group. Carvacrol could significantly improve insulin and high‐density lipoprotein cholesterol (HDL‐C) levels, and reduce concentrations of cholesterol, low‐density lipoprotein cholesterol (LDL‐C), triglycerides, blood urea nitrogen, urea, and creatinine (*p* < 0.001). In addition, total antioxidant capacity, GPx, and SOD levels were noticeably higher, and MDA level was significantly lower in the carvacrol–treated group compared with the diabetic group (*p* < 0.001). Furthermore, treatment with carvacrol significantly reduced mRNA expression levels of IL‐1β, IL‐12, ICAM‐1, VCAM‐1, and MCP‐1 compared with the diabetic group (*p* < 0.05). However, Nrf‐2 mRNA expression was significantly higher in the carvacrol–treated group than in the diabetic group (*p* < 0.001). Also, treatment with carvacrol could significantly improve glomerular diameters, diameters of urinary space, and number of glomeruli compared with the diabetic group (*p* < 0.001). Treatment with carvacrol could improve glycemic control, lipid profile, oxidative stress, and inflammatory markers in diabetic rats.

## Graphical abstract

Carvacrol administration is associated with improvements in lipid profiles, attenuation of oxidative stress, and modulation of inflammatory markers, including C‐reactive protein (CRP). The diagram summarizes systemic effects across major organs, highlighting potential protective roles against inflammation‐related oxidative damage and kidney injury.

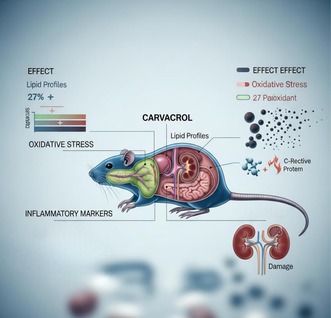

## 1 Introduction

Diabetes mellitus is characterized by elevated glucose levels due to inadequate insulin synthesis or action (Azgomi et al. 2024). Globally, the prevalence of diabetes is increasing; estimates suggest that 642 million people will have the disease by 2040, mostly in developing countries (Karimi et al. 2023). By 2030, there will likely be 439 million adults with diabetes worldwide, up from 285 million in 2010 (Shaw et al. 2010). Elevated blood glucose levels can harm numerous organs, including the eyes, kidneys, heart, and blood vessels (Kooshki et al. 2021).

Studies have demonstrated that oxidative stress, triggered by factors such as high blood sugar, insulin resistance, abnormal lipid levels, and inflammation, increases the risk of vascular complications in diabetes (Jazani et al. 2022). Increased protein kinase activity, disruption of antioxidant enzyme levels, and enhanced mitochondrial superoxide production during hyperglycemia can lead to oxidative stress from free radicals (Nasimi Doost Azgomi et al. 2021).

Diabetes is currently treated with insulin therapy and a variety of medicines such as thiazolidinedione inhibitors, α‐glucosidase inhibitors, and sulfonylureas (Joshi et al. 2015). Despite their effectiveness, these drugs may have significant adverse effects on the body. Certain mineral and herbal compounds have been shown to have beneficial effects on diabetes by either mimicking insulin's action or promoting the regeneration of non‐beta cells in the pancreas (Cefalu et al. 2012).

*Carvacrol* (2‐methyl‐5‐isopropylphenol) is a monoterpene phenolic compound widely distributed in the essential oils of aromatic plants and culinary herbs belonging to the Lamiaceae family, such as marjoram ( *Origanum majorana* L.), summer savory ( *Satureja hortensis* L.), and thyme ( *Thymus vulgaris* L.) (Zhao et al. 2020). Naturally occurring in these species, carvacrol contributes to their characteristic aroma and flavor and is commonly used as a natural food additive and preservative due to its strong antimicrobial and antioxidant capacities (Imran et al. 2022).

Studies have demonstrated that *Carvacrol* exerts potent immunomodulatory and anti‐inflammatory effects by modulating cytokine production and suppressing key signaling cascades, such as NF‐κB and the MAPK pathway (Imran et al. 2022; Zhao et al. 2020).

Additionally, *carvacrol* acts as an ion channel modulator, promoting the production of transient receptor potential vanilloid 3 (TRPV3) and transient receptor potential channel A1 (TRPA1) while suppressing the expression of transient receptor potential melastatin 7 (TRPM7) (Li et al. 2020). It has been demonstrated that inhibition of TRPM7 expression protects against high glucose‐induced neuronal death, and depletion of TRPM7 dramatically enhances insulin production in rat insulinoma cells (INS‐1) (Li et al. 2020). Research has also shown that TRPA1 helps maintain glucose homeostasis, and *carvacrol* can activate TRPV3 to mediate fibrosis and decrease the extracellular matrix (Li et al. 2020). *Carvacrol* modulates the PI3K/Akt signaling pathway to restore glucose transporter‐4 (GLUT4) membrane translocation (Hou et al. 2019). *Carvacrol* reversed cardiac hypertrophy, reduced cardiac fibrosis, and markedly improved blood glucose levels in mice with type 1 and type 2 diabetes mellitus (Habtemariam 2017).

Additionally, in high‐fat diet‐induced diabetic rats, *carvacrol* showed promise for its antihyperglycemic properties when combined with rosiglitazone (Hou et al. 2019). Furthermore, *carvacrol* attenuated diabetes‐associated cognitive deficits in rats (Habtemariam 2017). Although *carvacrol* has been reported to exert various pharmacological effects, its integrated impact on metabolic and inflammatory homeostasis, particularly in the context of diabetes‐induced kidney dysfunction, has not been clearly elucidated. Therefore, the present study aimed to investigate the effect of carvacrol on lipid profiles, oxidative stress, inflammatory markers, and renal injury in STZ‐induced diabetic rats.

## 2 Materials and Methods

### 2.1 Animal Preparation

This study utilized 30 male Wistar rats, weighing between 200 and 250 g each. The rats were fed a consistent diet for 2 weeks. They were kept in a controlled environment with a 12‐h light/dark cycle at 25°C. All protocols were adhered to, and experiments were conducted in strict accordance with the ethical principles and guidelines set forth by the Ethics Committee of Ardabil University of Medical Sciences (IR. ARUMS.AEC.1403.013).

By injecting 50 mg/kg of STZ dissolved in a 5 mM citrate buffer (pH 4.5), the experimental rats were made diabetic. Rats having blood sugar levels more than 250 mg/dL after 72 h were verified to have diabetes. There were three groups of 10 rats each. Group 1 consisted of healthy rats without any treatment (control group). Rats with diabetes receiving normal saline were part of Group 2. Rats with diabetes in Group 3 received 8 weeks of treatment with 250 mg/kg of *carvacrol*. Instead of receiving the STZ injection, the healthy control group received citrate. At the end of the eight‐week experimental period, all rats were anesthetized with a ketamine/xylazine (10/1 mg.kg^−1^) combination (Sigma Aldrich, Germany) before sample collection. Blood was collected via cardiac puncture for biochemical assessment of oxidative stress markers, glucose, insulin, lipid profile, and antioxidant parameters. After blood withdrawal, the kidneys were excised, rinsed with cold saline, snap‐frozen in liquid nitrogen, and stored at −80°C until biochemical and molecular analyses. Serum aliquots were also stored at −80°C for enzymatic activity assays. The treatment schedule was maintained consistently throughout the 8 weeks.

### 2.2 Biochemical Assays

#### 2.2.1 Lipid Profile, Glycemic Parameters, and Kidney Function Tests

BUN was quantified by an enzymatic colorimetric assay based on a coupled urease‐glutamate dehydrogenase reaction, which measures the rate of NADH consumption spectrophotometrically at 340 nm. Urea concentration was determined enzymatically using urease‐catalyzed hydrolysis of urea to ammonia, followed by quantification through a modified diacetyl (Fearon) reaction or enzymatic detection with spectrophotometric readouts. Creatinine levels were assessed using the Jaffe reaction, which involves the formation of a colored complex between creatinine and alkaline picrate, with absorbance measured at 490 nm Morsy et al. (2010).

Using commercial kits (MAK045), serum levels of cholesterol, triglycerides (TG), high‐density lipoprotein cholesterol (HDL‐c), and low‐density lipoprotein cholesterol (LDL‐c) were measured, and the results were expressed in mg/dL. Serum insulin levels were evaluated using an enzyme‐linked immunosorbent assay (ELISA) method with the Rat Insulin commercial kit (Thermo Fisher, Invitrogen, ERINS), and homeostatic model assessment for insulin resistance (HOMA‐IR) was calculated. The mean fasting plasma glucose and insulin concentrations were 4.38 mmol/L and 10.63 mIU/L, respectively. Using these values, the HOMA‐IR index was calculated to be 46.52 based on the following equation:

$$\text{HOMA}-\mathrm{IR}=\frac{\text{fasting insulin}\;\left(\mathrm{\mu U}/\mathrm{mL}\right)\times \text{fasting glucose}\;\left(\text{mmol}/L\right)}{22.5}$$

### 2.3 Assessment of Oxidative Stress Markers in Kidney Tissue

The tissue's oxidative stress was indicated by the malondialdehyde (MDA) level (Uchiyama and Mihara 1978). The TBA‐TCA‐HCl solution was prepared by dissolving 375 mg of TBA in 2 mL of HCl, then combining it with 100 mL of 15% trichloroacetic acid (TCA). The resultant precipitate was dissolved in a homogeneous solution at 50°C in a water bath. The result was a homogeneous mixture of 5.1% and 10% potassium chloride solutions. A pink‐orange solution was then produced by heating 1 mL of homogenized kidney tissue and 2 mL of the TBA‐TCA‐HCl solution in boiling water for 45 min. After cooling, the mixture was centrifuged for 10 min at 1000 rpm, and the absorbance was measured at 532 nm using a spectrophotometer (Biospect). The activities of superoxide dismutase (SOD) and glutathione peroxidase (GPx) were quantified using commercially available kits (RANSOD and RANSEL, Randox Laboratories Ltd., UK) in accordance with the protocols established (Dworzański et al. 2020; Weydert and Cullen 2010).

### 2.4 Quantitative Real‐Time PCR

Total RNA was extracted from kidney tissues of the control, diabetic, and carvacrol‐treated groups using TRIzol reagent (Sigma‐Aldrich, T9424). Next, the RNA concentration was determined using the NanoDrop (Thermo Fisher, USA). Subsequently, 1 μg of total RNA was used to produce complementary DNA (cDNA) according to the manufacturer's guidelines (BioFactTM). The primer sequences were blast‐blasted using the *NCBI Primer‐BLAST tool* before the experiment. A real‐time polymerase chain reaction (real‐time PCR) (Applied Biosystems StepOneTM) was carried out using particular primers for interleukin‐1 beta (IL‐1β), interleukin‐12 (IL‐12), intercellular adhesion molecule‐1 (ICAM‐1), vascular cell adhesion molecule‐1 (VCAM‐1), monocyte chemoattractant protein‐1 (MCP‐1), and nuclear factor erythroid 2‐related factor 2 (Nrf‐2) genes (Bioneer, Korea). Additionally, the 2^−ΔΔCt^ technique was used to obtain the results (Table 1).

**TABLE 1:** Sequence of gene primers for qRT‐PCR.

Genes	Forward	Reverse
MCP‐1	5′‐ATGCAGGTCTCTGTCACG‐3′	5′‐CTAGTTCTCTGTCATACT‐3′
*IL‐1β*	5′‐TGATGTTCCCATTAGACAGC‐3′	5′‐GAGGTGCTGATGTACCAGTT‐3′
*IL‐12*	5′‐GTGGGAGCTGGAGAAAGATG‐3′	5′‐TTGGTGCTTCACACTTCAGG‐3′
*ICAM‐1*	5′‐GTGATGCTCAGGTATCCATCCA‐3′	5′‐CACAGTTCTCAAAGCACAGCG‐3′
*VCAM‐1*	5′‐TTTGCAAGAAAAGCCAACATGAAAG‐3′	5′‐TCTCCAACAGTTCAGACGTTAGC‐3′
*Nrf‐2*	5′‐CACATCCAGACAGACACCAGT‐3′	5′‐CTACAAATGGGAATGTCTCTGC‐3′
*GAPDH*	5′‐CCGTGTTTCCTACCCCCAATG‐3′	5′‐CTTCACCAACCTTCTTGATGTCATC‐3′

### 2.5 Examination of Tissue

Tissue samples were preserved in 10% formalin, then dehydrated and embedded in paraffin. Subsequently, the samples were sectioned at 5 μm using a microtome. Each sample was then treated with H&E for precise identification. Some slides were used to assess histological changes, while others were used to evaluate the urinary tract and glomeruli.

### 2.6 Statistical Analysis

To analyze data, IBM SPSS Statistics 23 (IBM SPSS Statistics, Armonk, USA) was used. The data's normality was determined using the Kolmogorov–Smirnov test. The mean ± standard deviation (SD) of the data was displayed. One‐way analysis of variance (ANOVA) was used to analyze the data, and Tukey's test was used for post hoc comparisons. A *p*‐value of less than 0.05 was used to confirm statistical significance.

## 3 Results

### 3.1 The Effect of *Carvacrol* on Lipid Profile and Glycemic Indices

Table 2 shows that, although the levels of cholesterol, LDL‐c, HOMA‐IR, and TG were higher (*p* ≤ 0.001), the serum levels of insulin and HDL‐c were considerably lower (*p* < 0.001) in the diabetic group compared to the control group. Serum insulin and HDL‐c levels improved (*p* < 0.001) in *carvacrol*‐treated groups as compared to the diabetic group, but levels of cholesterol, LDL‐c, HOMA‐IR, and TG decreased (*p* < 0.001).

**TABLE 2:** Effect of carvacrol on lipid profile and glycemic indices.

Groups	Cholesterol (mg/dL)	HDL‐c (mg/dL)	LDL‐c (mg/dL)	TG (mg/dL)	Insulin (ng/ml)	HOMA‐IR
Control	53.71 ± 8.92	43.54 ± 5.27	24.14 ± 2.32	33.21 ± 3.73	2.49 ± 0.18	0.45 ± 0.04
Diabetic	84.57 ± 11.13†	21.92 ± 2.43†	41.27 ± 4.53†	70.05 ± 6.48†	1.67 ± 0.14†	1.21 ± 0.11
Treatment	66.42 ± 9.01*, †	35.24 ± 4.67*, †	30.12 ± 3.07*, †	45.57 ± 4.18*, †	2.12 ± 0.22*, †	0.76 ± 0.09*, †

*Note:* Mean ± SD are presented for the data. The symbol of † means the significant difference with the control group (*p* < 0.001), and the asterisk (*) shows the significant difference with the diabetic group (*p* < 0.001). HDL‐c, high‐density lipoprotein cholesterol; LDL‐c, low‐density lipoprotein cholesterol; TG, triglyceride; HOMA‐IR, homeostatic model assessment for insulin resistance. Treatment (Diabetes + Carvacrol). Treatment (Diabetes + Carvacrol).

Abbreviations: HDL‐c, high‐density lipoprotein cholesterol; HOMA‐IR, homeostatic model assessment for insulin resistance; LDL‐c, low‐density lipoprotein cholesterol; TG, triglyceride.

### 3.2 The Effect of *Carvacrol* on Serum Levels of Antioxidant Enzymes, BUN, Urea, and Creatinine

According to Table 3, diabetes led to a significant decrease in the activity of antioxidant enzymes, including TAC, GPx, and SOD, compared with the control group (*p* < 0.001). In addition, STZ‐induced diabetes led to a significant decrease in MDA levels compared with the control group (*p* < 0.001). However, treatment with carvacrol could significantly decrease MDA activity compared with the diabetic group (*p* < 0.001). In addition, TAC, GPx, and SOD levels were significantly higher in the carvacrol group (group 3) than in the diabetic group (*p* < 0.05). According to Table 3, the diabetic group had significantly higher blood urea nitrogen (BUN), urea, and creatinine levels than the control group. Additionally, treatment with *carvacrol* could significantly lower BUN, urea, and creatinine levels compared with the diabetic control group (*p* < 0.001).

**TABLE 3:** Effect of carvacrol on serum levels of antioxidant enzymes, BUN, urea, and creatinine.

Groups	SOD (U/mL)	MDA (nM)	GPx (U/ml)	TAC (U/mL)	BUN (mg/dL)	Urea (mg/dL)	Creatinine (mg/dL)
Control	3.11 ± 0.34	10.93 ± 1.73	25.38 ± 4.42	1.21 ± 0.24	4.14 ± 0.39	41.27 ± 4.12	0.32 ± 0.01
Diabetic	1.11 ± 0.57†	26.38 ± 2.54†	9.11 ± 2.21†	0.21 ± 0.57†	15.67 ± 2.05†	114.03 ± 9.47†	0.49 ± 0.02†
Treatment	1.71 ± 0.24*, †	17.81 ± 1.27*, †	18.73 ± 3.81*, †	1.01 ± 0.12*, †	7.27 ± 0.68*, †	59.84 ± 5.01*, †	0.27 ± 0.01*, †

*Note:* Mean ± SD is presented for the data. The symbol of † means the significant difference with the control group (*p* < 0.001), and the asterisk (*) shows the significant difference with the diabetic group (*p* < 0.001). Treatment (Diabetes + Carvacrol).

Abbreviations: BUN, blood urea nitrogen; GPx, glutathione peroxidase; MDA, malondialdehyde; SOD, superoxide dismutase; TAC, total antioxidant capacity.

### 3.3 Histological Assessment of Renal Tissue and Kidney Weight

**FIGURE 1:**
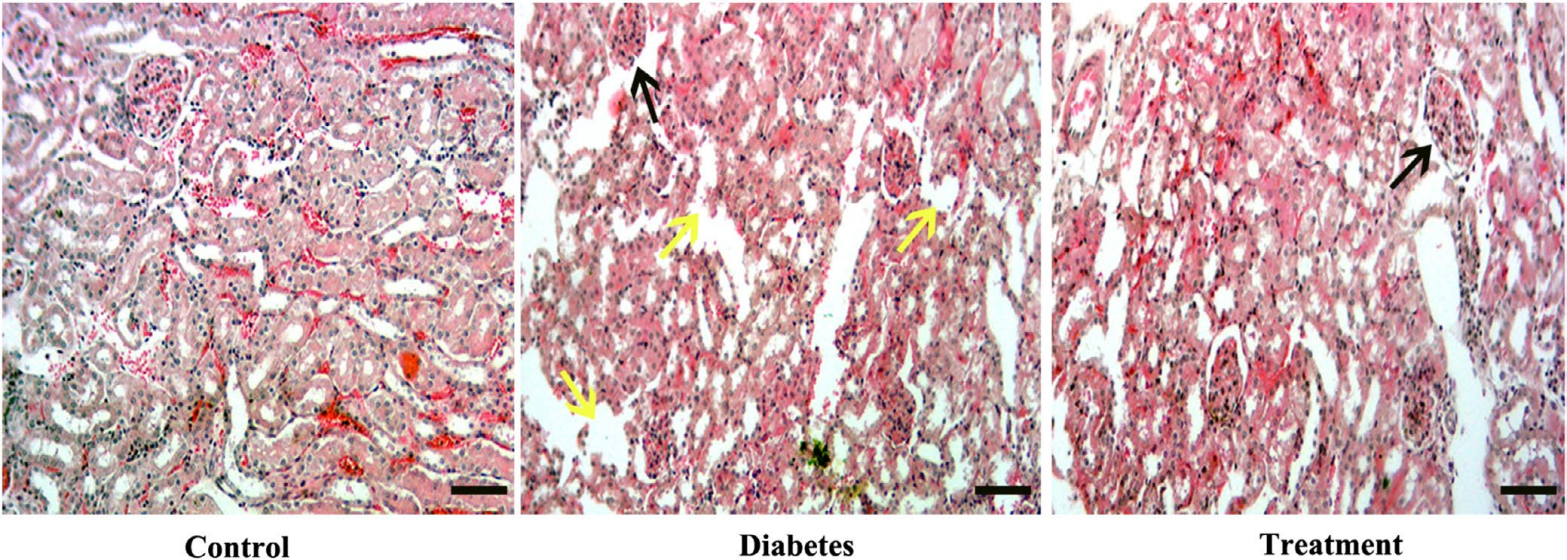
Histopathology of the kidney in all groups of the study. H&E staining in the control group showed normal kidney histology. In the diabetic group, the kidneys showed histopathological damage, including tubular detachment and rupture, as well as glomerular injury. However, in the treatment group (Diabetes+Carvacrol), the severe injuries caused by diabetes were reduced when carvacrol was administered. Black arrows show damaged glomerulus; yellow arrows show tubular damage. Scale bar: 20×.

The glomerular diameter in the diabetic group was substantially greater than that of the control group (*p* < 0.01). Treatment with *carvacrol* could significantly decrease the glomerular diameter compared with the diabetic group (*p* < 0.001). The diabetic group exhibited a substantial reduction in the number of glomeruli compared to the control group (*p* < 0.01). In the *carvacrol* group, the quantity of glomeruli was considerably increased (*p* = 0.01) compared to the diabetic group (Figure 1; Table 4). The diabetic group had significantly higher kidney weight than the control group. Treatment with *carvacrol* could significantly lower kidney weight compared with the diabetic group (*p* < 0.001; Table 4).

**TABLE 4:** Glomerular diameters, diameters of urinary spaces, and number of glomeruli in all groups of the study.

Groups	Kidney weight	Glomerular diameters	Diameters of urinary space	Number of glomeruli
Control	1.39 ± 0.13	25.9 ± 3.1	35.9 ± 5.2	32.20 ± 3.31
Diabetic	2.5 ± 0.13†	35.6 ± 10.7†	25.1 ± 4.81†	21.79 ± 1.43†
Treatment	1.65 ± 0.12*, †	24.1 ± 9.9*, †	34.1 ± 5.65*, †	25.04 ± 2.68*, †

*Note:* Mean ± SD is presented for the data. The symbol of † means the significant difference with the control group (*p* < 0.01), and the asterisk (*) shows the significant difference with the diabetic group (*p* < 0.001). Treatment (Diabetes + Carvacrol).

### 3.4 The Expression Levels of IL‐1β, IL‐12, ICAM‐1, VCAM‐1, Nrf‐2 and MCP‐1 Genes

**FIGURE 2:**
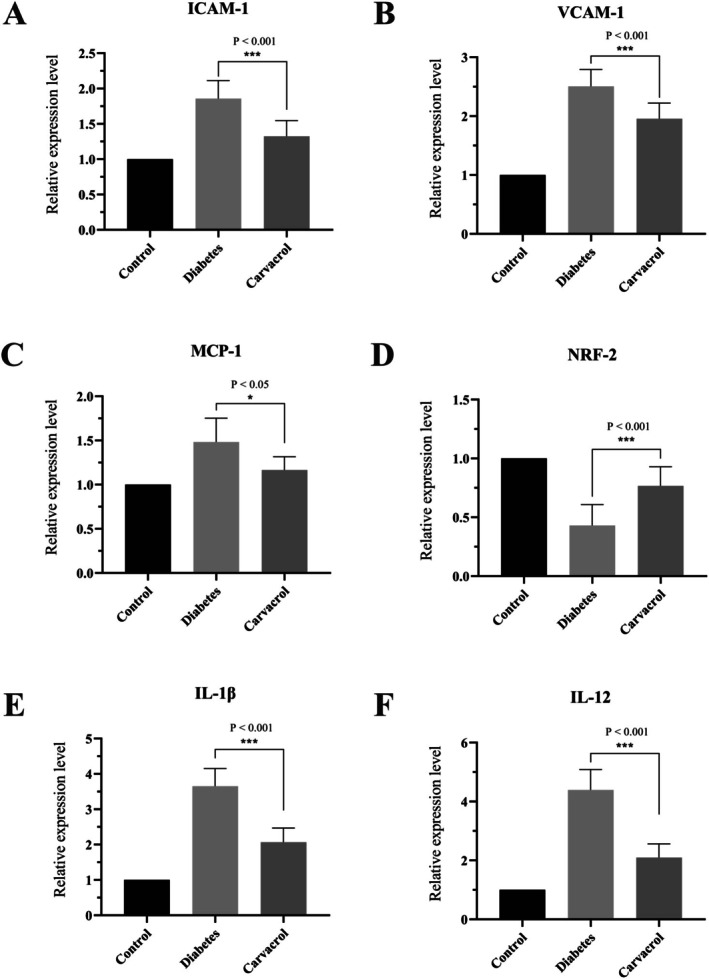
Gene expression: The figure shows the effect of carvacrol treatment on the mRNA expression levels of key inflammatory and oxidative stress‐related genes, including pro‐inflammatory cytokines IL‐1β and IL‐12, adhesion molecules ICAM‐1 and VCAM‐1, the antioxidant transcription factor Nrf‐2, and the chemokine MCP‐1. Real‐time PCR analysis was used to quantify gene expression changes. Statistically significant differences compared to the control group were determined by a *p*‐value less than 0.05 (*p* < 0.05). The results indicate that carvacrol modulates the inflammatory response by downregulating pro‐inflammatory genes and adhesion molecules, while potentially enhancing antioxidant defense via Nrf‐2 regulation. These molecular effects suggest that carvacrol has an anti‐inflammatory and protective role in the studied model.

The effects of carvacrol on the expression levels of genes associated with inflammation and antioxidants in renal tissue from diabetic rats were evaluated using RT‐qPCR. Figure 2 shows that STZ‐induced diabetes led to a statistically significant increase in mRNA expression levels of IL‐1β, IL‐12, ICAM‐1, VCAM‐1, and MCP‐1 compared with the control group (*p* < 0.05). Inversely, Nrf‐2 expression was significantly reduced in the diabetic group compared to the control group (*p* < 0.05). Treatment with carvacrol significantly reduced mRNA levels of IL‐1β, IL‐12, ICAM‐1, VCAM‐1, and MCP‐1 compared with the diabetic group (*p* < 0.05). However, carvacrol significantly increased Nrf‐2 mRNA expression compared with the diabetic control group (*p* < 0.001).

## 4 Discussion

The present study demonstrates that 8 weeks of *carvacrol* administration significantly ameliorated metabolic, oxidative, inflammatory, and renal alterations in STZ‐induced diabetic rats.

Although insulin and conventional hypoglycemic agents remain the cornerstone of diabetes mellitus management, their long‐term use is often limited by adverse effects and suboptimal efficacy, highlighting the ongoing need for safer and more effective therapeutic approaches (Hasanpour et al. 2018).

Long‐term use of chemical drugs often results in diminished effectiveness and can lead to several predictable side effects, such as increased hypoglycemic shock and fat storage. Currently, medicinal plants known for their antioxidant properties are increasingly used to treat various ailments, primarily due to their minimal side effects. *Carvacrol*, a monoterpenoid phenol found in the essential oils of various aromatic plants, such as oregano and thyme, has been extensively studied for its biological effects on animal health. STZ and alloxan are widely recognized as the primary agents for inducing diabetes in animals. These substances can damage pancreatic β‐cells by elevating oxidative stress, potentially leading to inappropriate changes in serum lipid and lipoprotein levels (Lenzen 2008). Additionally, under diabetic conditions, the liver plays a crucial role in the release of free fatty acids. This can result in an increased synthesis of cholesterol and phospholipids, as well as the secretion of certain lipoproteins into the bloodstream (Guzzaloni et al. 2000).

Our findings indicated that serum insulin and HDL‐C levels were elevated in the carvacrol group (Group 3) compared with the diabetic group (Group 2). Conversely, reductions in serum LDL‐C, TC, TG, and HOMA‐IR were observed. There is evidence that carvacrol has a strong anti‐hyperglycemic impact. It had a dose‐dependent effect on lowering fasting and random plasma glucose levels. Mice with diabetes treated with carvacrol showed significant improvements in their tolerance to glucose (Li et al. 2020). The anti‐hyperglycemic effect of carvacrol is particularly notable, as it not only reduces fasting plasma glucose levels but also improves glucose tolerance (Li et al. 2020). Treatment with *carvacrol* has also been linked to a significant reduction in plasma TG levels, which is a favorable effect in diabetes, considering the prevalent lipid abnormalities in diabetic conditions. It is worth noting that the research did not detect a statistically significant difference in plasma TC and insulin levels between diabetic mice treated with *carvacrol* and diabetic mice treated with vehicle (Hashemipour et al. 2013). By regulating glucose metabolism and increasing the activity of hepatic enzymes, namely hexokinase (HK), 6‐phosphofructokinase (PFK), and citrate synthetase (CS), carvacrol exerts an anti‐hyperglycemic effect. Since these enzymes are essential to glucose metabolism, higher enzyme activity may improve glucose regulation. It has been demonstrated that carvacrol significantly affects insulin resistance. In a study using mice with type 2 diabetes, carvacrol was found to improve insulin resistance and blood glucose levels (Zhao et al. 2021). However, another study conducted on STZ‐induced diabetic mice showed no substantial change in the plasma level of insulin between diabetic mice and *carvacrol*‐treated diabetic mice (Li et al. 2020). Further research is needed to fully understand its mechanisms and to evaluate its efficacy and safety in human subjects.

In the context of lipid profile, previous studies suggest that carvacrol may influence lipid metabolism and positively impact animal lipid profiles. A comprehensive review by Khazdair et al. (2024) highlighted the protective effects of *carvacrol* on oxidative stress, lipid profiles, hypertension, and cardiac dysfunction. The review synthesized data from multiple studies, indicating that *carvacrol* can alter lipid profiles by reducing heart rate and blood pressure, and improving oxidative stress markers. It also suggested that carvacrol's pharmacological effects on cardiovascular disease might be mediated by its antioxidative, antiapoptotic, and anti‐inflammatory properties. Another study focused on the molecular properties of *carvacrol* and its therapeutic potential, emphasizing its antimicrobial, anti‐inflammatory, antitumor, and anti‐hepatotoxic activities. The study discussed *carvacrol's* physicochemical properties, such as its partition coefficient and pKa, which are indicative of its ability to alter pharmacotherapeutic profiles (Ahmad et al. 2021). Galvão et al. (2020) discussed how increasing *carvacrol* content in mixtures could decrease the crystallinity of solid bulks, which is postulated as an advantage for increasing the loading capacity of lipid carriers. This finding is significant as it suggests *carvacrol's* potential role in drug delivery systems, particularly for lipid‐based formulations. The mechanisms by which *carvacrol* affects lipid profiles are not fully understood, but it is believed to be related to its antioxidant and anti‐inflammatory properties. *Carvacrol's* ability to modulate gut microflora and improve digestion and absorption of nutrients might also play a role in its effects on lipid metabolism (Li et al. 2020). Moreover, *carvacrol's* impact on lipid profiles may extend beyond just cholesterol management. It could also influence other aspects of lipid metabolism, such as fatty acid synthesis and breakdown, which are crucial for maintaining the overall health and productivity of animals.

In the present study, treatment with *carvacrol* significantly increased TAC and SOD levels, while significantly reducing MDA levels in diabetic rats compared with the untreated diabetic group. It has been discovered that *carvacrol* alters the expression and function of antioxidant enzymes. In a study on the pathogenic fungus Candida auris, *carvacrol* was found to greatly boost the activity and gene expression of the main antioxidant enzymes (Ismail et al. 2022). In another study, carvacrol supplementation in the feed was found to increase antioxidant enzyme activities in broiler chickens (Hashemipour et al. 2013). *Carvacrol's* phenolic structure is thought to be responsible for its antioxidant qualities. *Carvacrol's* phenolic group possesses strong antibacterial and antioxidant properties. Because of its benzene ring and methyl and isopropyl substituents, it has a hydrophobic character that facilitates its binding to DNA's guanine (Mondal et al. 2021). In addition to its antioxidant properties, carvacrol also exerts strong anti‐inflammatory effects by reducing levels of pro‐inflammatory cytokines. This dual action, with antioxidant and anti‐inflammatory properties, makes carvacrol a promising compound for therapeutic applications (Mączka et al. 2023). Administering *carvacrol* significantly improved renal histological alterations and antioxidant protein levels. *Carvacrol* inhibits the epithelial‐to‐mesenchymal transition process in TGF‐β1‐stimulated renal tubular epithelial cells (NRK 52E cells), according to findings from an in vitro investigation (Ram et al. 2022). Strong anti‐inflammatory effects of *carvacrol* have been claimed to be achieved via inhibiting polyunsaturated fatty acid peroxidation (Gunes‐Bayir et al. 2022). It has been suggested that *carvacrol* has potent anti‐inflammatory effects by halting polyunsaturated fatty acid peroxidation (Mączka et al. 2023). In a study, *carvacrol* was administered to rats exposed to cadmium as a major environmental pollutant (Yesildag et al. 2022), and the findings showed that *carvacrol* increased levels of SOD, GPx, and catalase, and lowered cadmium‐induced levels of MDA (Yesildag et al. 2022). In the present investigation, carvacrol significantly decreased the mRNA levels of inflammatory markers, including IL‐1β, IL‐12, ICAM‐1, VCAM‐1, and MCP‐1, in diabetic rats. *Carvacrol* has been shown to reduce inflammation by inhibiting NF‐κB and MAPK, thereby reducing the production of pro‐inflammatory cytokines. Furthermore, *carvacrol* could lower oxidative stress, inflammation, and apoptotic impact in *N*‐methyl‐*N*′‐nitro‐*N*‐nitrosoguanidine (MNNG) induced gastric carcinogenesis in Wistar rats (Gunes‐Bayir et al. 2022). Additionally, *carvacrol* could reduce acute kidney damage caused by acetaminophen by inhibiting the biochemical processes involved in oxidative stress and apoptosis (Najafizadeh et al. 2022). A systematic review and meta‐analysis conducted by de Carvalho et al. (2020) showed that carvacrol was beneficial in reducing key inflammatory markers, including MDA, IL‐4, IL‐8, and IL‐1β. Nevertheless, the research revealed that carvacrol had no discernible effect on IL‐6 and TNF‐α, most likely due to the studies' variability and poor methodology. Carvacrol was shown to prevent acrylamide‐induced oxidative and inflammatory liver damage in another study. It is well known that acrylamide can be hepatotoxic due to its effects on inflammatory and oxidative processes. The results of the investigation demonstrated that carvacrol both reduced histological damage and prevented the biochemical alterations induced by acrylamide (Cerrah and Suleyman 2023). *Carvacrol* has also been demonstrated to block the synthesis of E2, F1, and F2 prostaglandins as well as neutrophil elastase. Moreover, it can inhibit COX‐2 activity (Alizadeh et al. 2017).

In the present study, serum levels of BUN, urea, and creatinine were significantly reduced in *carvacro*l‐treated diabetic rats compared with the untreated diabetic group, indicating an improvement in renal function. Previous studies have demonstrated the protective effects of carvacrol against nephrotoxic injury (Ahmadvand et al. 2016). investigated the impact of carvacrol co‐administration in nephrotoxic rats induced by gentamicin. In their 12‐day study, one group received gentamicin (GS) at 100 mg/kg daily, while another group was treated with GS plus carvacrol at 74 mg/kg. Histopathological examination revealed that carvacrol significantly decreased leukocyte infiltration and tubular necrosis compared to untreated nephrotoxic controls. Biochemically, carvacrol administration substantially reduced serum urea and creatinine levels, indicating improved renal function (Ahmadvand et al. 2016). These results imply that carvacrol improves renal function tests in the nephrotoxic group by reducing tubular necrosis and loss of leukocyte infiltration. To fully understand the mechanisms of action and potential therapeutic uses of carvacrol in kidney function and health, further research is needed.

Diabetes is a major global health challenge, making it essential to investigate effective treatments such as carvacrol for alternative therapeutic strategies. Using a STZ‐induced diabetic rat model enables controlled trials that may provide insights into the human condition, while focusing on oxidative stress and inflammatory markers could shed light on the mechanisms underlying the potential benefits of carvacrol. However, limitations such as the translational gap between animal and human models, the lack of clinical data, potential confounding factors, and the need to distinguish between short‐ and long‐term effects must be considered to fully understand its therapeutic implications.

## 5 Conclusion

Based on the results of the present study, carvacrol improves diabetic kidney damage by enhancing antioxidant defense and reducing the expression of inflammatory factors, including IL‐1β, IL‐12, ICAM‐1, VCAM‐1, and MCP‐1. This beneficial role is mediated by increasing the activities of SOD, GPX, and TAC and reducing MDA levels in the treatment group compared to the diabetic group. Also, a significant decrease in biochemical parameters, including BUN, creatinine, and urea, was observed in the treatment group compared with the diabetic group. In addition, histopathological changes caused by renal damage were improved in animals treated with carvacrol compared to the untreated diabetic group.

## Author Contributions

V.S.D., V.A., H.T., E.F., R.M., M.A.V., and H.S. created the research. Data screening and literature searches were carried out by V.A., H.T., and M.A.V. V.S.D. and H.S. independently extracted data and evaluated its quality. The text was written by V.S.D., V.A., and H.T. after data interpretation. The study was headed by M.A.V. The final manuscript was read and approved by all writers.

## Funding

This study was financially supported by Ardabil University of Medical Sciences (Project code: 402000722).

## Ethics Statement

All experimental procedures involving animals were approved by the Ethics Committee of Ardabil University of Medical Sciences (Approval No. IR.ARUMS.AEC.1403.013) and were conducted in accordance with international guidelines for the care and use of laboratory animals.

## Consent

The authors have nothing to report.

## Conflicts of Interest

The authors declare no conflicts of interest.

## Data Availability

Data will be made available on request.
